# Association between Diet Quality and Index of Non-Alcoholic Steatohepatitis in a Large Population of People with Type 2 Diabetes: Data from the TOSCA.IT Study

**DOI:** 10.3390/nu14245339

**Published:** 2022-12-15

**Authors:** Marilena Vitale, Giuseppe Della Pepa, Giuseppina Costabile, Lutgarda Bozzetto, Paola Cipriano, Stefano Signorini, Valerio Leoni, Gabriele Riccardi, Olga Vaccaro, Maria Masulli

**Affiliations:** 1 Department of Clinical Medicine and Surgery, University of Naples Federico II, 80131 Naples, Italy; 2 Laboratory of Clinical Biochemistry, Hospital Pius XI of Desio, ASST-Brianza, 20833 Desio, Italy; 3 Department of Medicine and Surgery, University of Milano-Bicocca, 20216 Monza, Italy

**Keywords:** type 2 diabetes, NASH, micronutrients, macronutrients, dietary habits, foods groups, Mediterranean diet, dietary patterns

## Abstract

Background: There are still open questions with respect to the optimal dietary treatment in patients with type 2 diabetes (T2D) and coexisting non-alcoholic steatohepatitis (NASH). The aim of this study is to investigate, in patients with T2D, the association between NASH, dietary component intake, food groups and adherence to the Mediterranean diet. Methods: Cross-sectional analysis of 2026 people with T2D (1136 men and 890 women). The dietary habits were assessed with the European Prospective Investigation into Cancer and Nutrition (EPIC) questionnaire. NASH was identified by the Index Of NASH (ION). Based on the cluster analysis two dietary patterns were identified: the NASH and the NO-NASH pattern. Results: The macronutrient composition of the diet was similar in the two patterns. However, the NASH pattern compared with the NO-NASH pattern was characterized by a significantly lower content of fibre (*p* < 0.001), β-carotene (*p* < 0.001), vitamin C (*p* < 0.001), vitamin E (*p* < 0.001), polyphenols (*p* = 0.026) and antioxidant capacity (*p* < 0.001). With regard to food consumption, the NASH pattern compared with NO-NASH pattern was characterized by higher intake of rice (*p* = 0.021), potatoes (*p* = 0.013), red (*p* = 0.004) and processed meat (*p* = 0.003), and a lower intake of wholegrain bread (*p* = 0.019), legumes and nuts (*p* = 0.049), vegetables (*p* = 0.047), fruits (*p* = 0.002), white meat (*p* = 0.001), fatty fish (*p* = 0.005), milk and yogurt (*p* < 0.001). Conclusions: NO-NASH dietary pattern was characterized by a food consumption close to the Mediterranean dietary model, resulting in a higher content of polyphenols, vitamins, and fibre. These finding highlight the potential for dietary components in the prevention/treatment of NASH in people with T2D.

## 1. Introduction

Non-alcoholic fatty liver disease (NAFLD) represents the most common liver disease worldwide affecting approximatively 20–30% of the general population [1]. The histopathological and clinical abnormalities of NAFLD spectrum ranges from the accumulation of triglycerides in the liver, i.e., non-alcoholic fatty liver (NAFL), to the inflammation and cellular damage of the hepatocytes, i.e., non-alcoholic steatohepatitis (NASH), that may progress to liver fibrosis and advanced cirrhosis [2]. The most serious clinical manifestations of NAFLD, i.e., NASH and cirrhosis, have very recently become the fastest growing indications for liver transplantation in western countries, heavily impacting on patient health, economic aspects and quality of life [3].

Interestingly, NAFLD is strictly associated with the features of metabolic syndrome such as obesity, type 2 diabetes (T2D), dyslipidaemia and hypertension [4]. In particular, the association between NAFL/NASH and T2D is well established and there appears to exist an intricate interrelationship whereby the existence of one drives progression to the other. T2D seems to be the most important risk factor for NAFLD and the most important clinical predictor of the advanced forms of NAFLD [5,6]. On the other hand, NAFLD is associated with a worse metabolic profile [7,8] and a higher prevalence of microvascular and macro-vascular complications of diabetes, independently of other known risk factors [9,10,11]. From an epidemiological point of view, it is not surprising that there is a high prevalence of NAFL and NASH in T2D, estimated at 55–70% and 20–40%, respectively [12], and higher in T2D with obesity [13].

Although liver biopsy represents the gold standard for the diagnosis of NASH, it is not feasible in large epidemiological studies. Several indices, based on non-invasive measures easily performed in clinical practice, have been proposed for the diagnosis of NASH [14], although none of these has been validated in people with diabetes. Among others, we used the Index Of NASH (ION), an algorithm constructed from the combination of triglycerides, visceral obesity, alanine aminotransferase (ALT) and Homeostatic Model Assessment (HOMA-IR), and validated against liver biopsy in an obese population sharing several metabolic and clinical features with T2D (i.e., obesity, excess of visceral fat, insulin resistance and high prevalence of NASH) [15]; in this population, the ION has shown a good diagnostic accuracy (AUC = 0.88 [95%CI 0.82–0.95]), with a sensitivity of 92% and a specificity of 60% [15].

No pharmaceutical approaches for NAFLD have been approved to date, and the cornerstone in the prevention and treatment of NAFLD and its severe forms is represented by lifestyle modifications, including diet-related factors [14,16]. Some attempts have been made to clarify the association between dietary components and NAFLD in the general population. Outside the context of clinical trials, epidemiological studies show that high glycaemic index foods and intake in saturated fats and simple sugars—fructose in particular—are associated with a higher prevalence in NAFLD [17,18,19,20,21]; whereas the intake of *n*-3 and *n*-6 polyunsaturated fatty acids (PUFA), monounsaturated fatty acids (MUFA), and fibre [22] appears to be associated with a lower prevalence of NAFLD [18,21,22]. Among micronutrients, intake of vitamins [23] and polyphenols is associated with a lower prevalence of NAFLD and might beneficially impact on the progression from NAFL to NASH [24].

In individuals with coexisting T2D and NAFL/NASH, hypocaloric diets promoting a weight loss of 7–10% are effective in improving metabolic parameters of both diseases [14], but they are not feasible in the long term and the optimal dietary model for people with T2D and NASH, not subjected to caloric restriction, remains ill-defined [25,26]. Nutritional guidelines for the treatment of diabetes recommend 45–60% of carbohydrates, selecting those with a low glycaemic index and high in fibre, 25–35% of fats, preferring MUFA and PUFA, 15–20% of proteins, and limiting/avoiding the intake of free sugars, sugar-sweetened beverages and added fructose [26,27]. These recommendations are designed with the main focus on correction of hyper-glycaemia; furthermore, the patient’s compliance is generally low/very low. Indications based on food consumption, rather than on nutrients, may improve adherence, but evidence regarding the possible association between habitual food consumption and NASH in T2D is lacking.

The aim of this study is to investigate in a large population of patients with T2D the association of habitual diet (i.e., diet composition and food consumption) with NASH, in order to expand knowledge on the potential for dietary components in the prevention/treatment of NASH in people with T2D.

## 2. Materials and Methods

### 2.1. Study Design and Population

We conducted a cross sectional study within the framework of the TOSCA.IT study (NCT00700856), a randomized controlled trial designed to evaluate the effects of sulfonylurea or pioglitazone, in add-on to metformin, on cardiovascular events in people with T2D. For the aim of the present study, we used data collected at baseline, prior to randomization to the study treatments.

The study participants were people with T2D, aged 50–75 years, on stable treatment with a full dose of metformin (2–3 g per day), and with a glycated hemoglobin (HbA1c) between 7% and 9%. Participants with incomplete data sets, those with alcohol intake exceeding 30 g/day if men and 20 g/day if women, or taking *n*-3 supplements were excluded from the analyses [28]; other exclusion criteria were severe hepatic dysfunction (plasma ALT values > 2.5 times the upper normal limit), serum creatinine > 1.5 mg/dL, history of congestive heart failure, (NYHA class I or higher), ulcer or gangrene of the lower extremities, cancer, substance abuse, and any health problem requiring special dietary treatments. Details of the study protocol have been published [29,30]. NASH was defined based on ION ≥ 50. To identify the association of the habitual diet with NASH, dietary patterns associated with ION ≥ 50 or <50 were derived using the K-means cluster analysis by which the sample population is classified into homogenous groups presenting different characteristics using a specific variable as the comparison criterion, in our case the ION. To perform this analysis, firstly the 248 food items were categorized into 59 food groups based on their similarity in term of nutrient composition. Then, the K-means clustering method was performed and the algorithm utilized to identify within each cluster the smallest variation. Two clusters were produced using a non-hierarchical K-means clustering method, with the random seed and 10 iterations to refine and optimize the classifications, and participants were grouped according to Euclidean distances. Two clusters were identified, one associated with an ION ≥ 50, the other associated with an ION < 50, respectively defined in the text and tables as cluster NASH or cluster NO-NASH. The anthropometric, metabolic and nutritional variables were compared in these two patterns. The study protocol has been approved by the Ethics Review Committee of the Coordinating Center and of each participating center. All participant provided written informed consent before entering the study.

### 2.2. Assessment of Anthropometric and Laboratory Parameters

Body weight was measured by mechanic balance (Seca 721), height with bar-altimeter, waist and hip circumference using an anelastic meter. Waist circumference was measured halfway between the lower ribs and the iliac crest and hip circumference was measured at the largest point around the buttocks. All measures were taken with an accuracy to the nearest 0.1 kg and 0.1 cm, respectively, and with the patient in light clothing and no shoes. Body mass index (BMI) was calculated as weight (kg)/height (m^2^). Blood pressure was measured in the sitting position by a standard protocol. Blood samples were obtained in the morning after an overnight fast and all biochemical parameters were analyzed in a central laboratory. Plasma glucose, total cholesterol, HDL-cholesterol, triglycerides, liver enzymes—ALT, aspartate aminotransferase (AST), and gamma-glutamyl-transpeptidase (GGT)—and high sensitivity *C*-reactive protein were detected by standard methods. LDL-cholesterol was calculated according to the Friedewald equation for triglyceride values < 400 mg/dL. HbA1c was measured with high liquid performance chromatography standardized according to IFCC. Plasma insulin was detected by ELISA (DIAsource ImmunoAssays S.A., Nivelles, Belgium) on a Triturus Analyser (Diagnostics Grifols, S.A., Barcelona, Spain). Insulin resistance was evaluated by the HOMA method calculated as follows: fasting glucose (mg/dL) × fasting insulin (μU/mL)/405.

### 2.3. Assessment of Dietary Intake, Food Consumption and Adherence to the Mediterranean Diet

The evaluation of eating habits was performed through the Italian version of the European Prospective Investigation into Cancer and Nutrition (EPIC) questionnaire, a validated food frequency questionnaire (FFQ) for the assessment of dietary habits in large epidemiological studies [31,32]. Details have been reported elsewhere [33,34]. Briefly, the FFQ is based on 248 items for which the respondent has to report (1) the absolute frequency of consumption in terms of per day, week, month, or year, and (2) the quantity by selection of pictures showing the portion size as small, medium and large, with additional quantifiers (i.e., “smaller than the small portion” or “between the small and medium portion”, etc…). Incomplete questionnaires and questionnaires with energy intake less than 800 or greater than 5000 kcal/day were excluded. A specific software (Nutrition Analysis of food frequency questionnaire—FFQ) was used to obtain the average daily amounts of foods (g/day) [31,32] and the energy and nutrient composition of the habitual diet [35,36]. The intake of polyphenols was evaluated using the USDA database [37] in combination with the Phenol-Explorer^®^database [38], as reported in more detail elsewhere [33,34]. In order to evaluate the adherence to the Mediterranean Diet, the relative Mediterranean Diet (rMED) score was used [39] as described in a prior publication [40]. Briefly, the average daily intake of fruits, vegetables, cereals, legumes, fish, olive oil, meat and meat products, dairy products and alcohol was divided in tertiles and assigned a score of 0, 1 or 2 to the first, second or third tertile, respectively, for the groups fitting the Mediterranean model, whereas for meat and dairy products, we assigned a score of 0, 1 or 2 to the third, second and first tertile, respectively. Regarding alcohol intake, 2 points for moderate intake (i.e., 5–25 g/day for women and 10–50 g/day for men, respectively) and 0 points for a consumption at or below the sex-specific range were assigned. The rMED score ranged from 0 to 18.

### 2.4. Assessment of Non-Alcoholic Steatohepatitis

NASH was calculated as indirect index by the ION according to the following formula: 1.33 waist to hip ratio + 0.03 × triacyclglycerols (mg/dL) + 0.18 × ALT (U/l) + 8.53 × HOMA − 13.93 for men; 0.02 × triacyclglycerols (mg/dL) + 0.24 × ALT (U/l) + 9.61 × HOMA − 13.99 for women. NASH was identified by an ION score of ≥50 [15].

### 2.5. Statistical Analysis

Data are presented as means ± standard deviation, frequencies and percentages, as appropriate. The *t*-test for independent samples was used to compare group means; for skewed variables, log transformed values were used. The χ^2^ test vas used to compare frequencies. A *p*-value < 0.05, two-tailed, was considered significant and all analyses were conducted with the SPSS Statistics software 28.0 (SPSS/PC; IBM, Armonk, NY, USA).

## 3. Results

The study population consists of 2026 people with T2D (1136 men and 890 women) with a mean age of 62.1 ± 6.5 years, a mean BMI of 30.3 ± 4.5 kg/m^2^ and a mean duration of diabetes of 8.5 ± 5.7 years. The prevalence of NASH according to the ION was 32%.

In Table 1 are reported the general characteristics and the cardio-metabolic profile for participants in the two clusters. The BMI, waist and hip circumference, waist/hip ratio, systolic and diastolic blood pressure, HbA1c, fasting plasma glucose and insulin, HOMA-IR, plasma LDL-cholesterol, plasma triglycerides, and liver enzymes were significantly higher among people in the cluster NASH as compared with those in the cluster NO-NASH, while age, diabetes duration and plasma HDL-cholesterol values were significantly lower. The proportion of smokers was similar in the two clusters, and a high proportion of the population was taking lipid lowering medications (62%) and/or antihypertensive medications (93%), with no significant differences between the two clusters.

In Table 2 are reported the energy intake and the nutrient composition of the diet in the participants in the two clusters. A significantly lower intake of energy, fibre, vitamin C, ß-carotene, vitamin E and polyphenols, was observed in the cluster NASH; accordingly the antioxidant capacity of the diet, estimated as Trolox Equivalent Antioxidant Capacity (TEAC), Total Radical-Trapping Antioxidant Parameter (TRAP), Ferric Reducing-Antioxidant Power (FRAP) was significantly lower. No differences were detected for the other components of the diet between the groups.

Coherent with these findings, the rMED score, an indicator of the overall quality of the adherence to the Mediterranean dietary pattern, was significantly lower in the cluster NASH (Table 3). This data was due to significant differences in individual foods and food group consumption (Table 3). People in the cluster NASH showed a significantly higher consumption of pasta, rice, potatoes, total meat, red meat, processed meat and a lower consumption of wholegrain bread, legumes and nuts, vegetables, fruits, white meat, fatty fish, total dairy products, milk and yogurt. The consumption of total cereals, white bread, total fish, lean fish, cheese, eggs, vegetable oils and fats from animal origin was not significantly different between the groups.

## 4. Discussion

To the best of our knowledge, this is the first large cross-sectional study evaluating the association between NASH and habitual diet in a well-characterized sample of adults with T2D.

The prevalence of NASH in the study population was high (32%) and in line with recent epidemiological data, as was the more adverse cardiovascular risk factors profile observed in the cluster NASH [12]. The association between nutrient composition of the diet and NAFLD, particularly NAFL, has been reported in prior epidemiological studies [41], but less is known regarding the association with more advanced stages of NAFLD (i.e., NASH) and no data are available on the association between habitual diet and NASH in patients with T2D. Here, we provide data on this association, thus expanding current knowledge.

The first remarkable finding is that the nutrient distribution was largely similar in the NASH or NO-NASH cluster. This is at variance with studies assessing the relation between NAFDL and diet composition in non-diabetic people which describe higher intakes of cholesterol, saturated fat [42,43] and added sugars [44,45] in individuals with NAFLD in comparison with matched controls without NAFLD. This inconsistency might be related to differences in the study design; in fact, we studied a population consisting of patients with T2D regularly attending diabetes clinics and who, therefore, may have restricted the consumption of the above-mentioned items as a result of medical advice, thus diluting the association.

The second relevant finding of this study is the association between fibre, micronutrient intake and NASH. Interestingly, despite the composition in macronutrients being similar between groups, the overall quality of the diet was very different between groups; with this regard, the intake of fibre, vitamins and polyphenols was significantly lower in the cluster NASH. These results are in line with epidemiological and clinical studies performed in patients without T2D [23,46,47]. All together, available evidence suggests that vitamins and polyphenols might prevent the advance of steatosis to NASH, probably restoring oxidative stress and reducing the transcription of the pro-inflammatory cytokines, which are the main drivers in the progression from NAFL to NASH [24,48,49]. Furthermore, dietary fibre might positively influence NAFL by acting on postprandial metabolic state, decreasing glucose absorption with a consequent reduction of the hepatic influx of glucose and de-novo lipo-genesis [22]. In addition, dietary fibre might stimulate a healthy gut microbiota, consequently decreasing the development of tissue inflammation and liver injury that led to NASH [50,51] and expanding also to NASH the interest in bioactive food compounds for T2D.

In terms of food groups, the cluster NASH was characterized by a lower consumption of whole grain bread, legumes and nuts, vegetables, fruits, fatty fish, milk and yogurt and a higher consumption of pasta, rice, potatoes, red meat and processed meat, providing data on the relation between food groups, NASH and T2D in line with those available for people without diabetes [43,52,53].

These data indicate that overall the NO-NASH dietary pattern is close to the Mediterranean dietary model, contributing to the growing evidence suggesting this model as the reference nutritional pattern to prevent and treat NAFLD [14,54], also in people with T2D. The beneficial effects of the Mediterranean diet on NAFLD might be related to dietary components such as dietary fibre, polyphenols and vitamins, that lead to the enhancement in the most important risk factors of NAFLD, such as BMI, insulin resistance and serum triglycerides [54], which are also key pathogenic factors for the development of T2D and major determinants of blood glucose control once diabetes has developed.

To our knowledge, this is the first epidemiological study on a large population of patients with T2D to evaluate the association between NASH and different dietary components and food groups, in a real-life setting.

There are still many open questions with respect to dietary treatment in individuals with both NASH and T2D. Importantly, in spite of the intimate relation between NAFLD and T2D, there are few nutritional intervention trials in which patients with coexisting T2D and NASH have been included. Therefore, it is unclear whether results from the numerous trials performed in patients with NASH and without T2D can be generalized to patients with both diseases. This study, by including a detailed analysis of both vegetable-based and animal-based foods, raises hypotheses on the overall dietary approach for NASH in people with diabetes, which could require confirmation in future large randomized-controlled trials.

Some limitations of the study must be acknowledged. First, the causal relationship between dietary components, Mediterranean dietary score and NASH cannot be proven due to the cross-sectional study design. Second, potential confounding from unmeasured lifestyle factors, such as physical activity level, might exist. Furthermore, data regarding dietary habits were collected only once and, consequently, could be prone to seasonal fluctuation and recall bias. Finally, NASH was detected by an indirect index currently accepted by NAFLD guidelines [16,55]. This index, although not specifically developed for people with diabetes was, however, validated in people with obesity who share several metabolic and clinical features with T2D (obesity, excess of visceral fat, insulin resistance and high prevalence of NASH), [15].

These limitations are counterbalanced by several strengths: a large sample size, a well-defined population of patients with T2D studied within the context of real-life clinical practice, the collection of nutritional and clinical data according to standard methods and biochemical measurements performed in a centralized laboratory.

## 5. Conclusions

In conclusion, this is one of the first epidemiological studies to investigate the dietary correlates of NASH in free living people with T2D focusing on foods and food groups. The results provide insights regarding habitual food consumption and dietary components as correlates of NASH in people with coexisting diabetes, showing that the NO-NASH dietary pattern is characterized by a higher intake of whole grain-based foods, legumes, nuts, fruits, vegetables, fatty fish, milk and yogurt, translating into a higher intake of polyphenols, vitamins and fibre and a higher Mediterranean dietary score. These findings expand current knowledge by highlighting the potential for dietary components in the prevention/treatment of NASH in people with T2D.

## Figures and Tables

**Table 1:** General characteristics and metabolic profile in the clusters NASH or NO-NASH.

	Cluster NASH (n. 642)	Cluster NO-NASH (n. 1384)	*p*-Value
Age (years)	62 ± 7	63 ± 6	0.006
Smoking (%)	34.4	32.6	0.209
Diabetes duration (years)	8 ± 5	9 ± 6	0.004
BMI (kg/m^2^)	32 ± 4	29 ± 4	<0.0001
Waist circumference (cm)	108 ± 11	102 ± 11	<0.0001
Hip circumference (cm)	109 ± 11	105 ± 10	<0.0001
Waist/Hip ratio	0.99 ± 0.98	0.96 ± 0.91	0.003
Systolic blood pressure (mm/Hg)	136 ± 16	134 ± 15	0.004
Diastolic blood pressure (mm/Hg)	81 ± 9	80 ± 9	0.002
HbA1c (%)	7.8 ± 0.5	7.6 ± 0.5	<0.0001
Plasma Glucose (mg/dL)	185 ± 39	159 ± 32	<0.0001
Plasma Insulin (μU/mL)	23.5 ± 12.2	9.4 ± 3.6	<0.0001
HOMA-IR	10.7 ± 7.0	3.6 ± 1.4	<0.0001
Plasma HDL-cholesterol (mg/dL)	43 ± 12	48 ± 12	<0.0001
Plasma LDL-cholesterol (mg/dL)	104 ± 30	101 ± 36	0.045
Plasma Triglycerides (mg/dL)	184 ± 114	137 ± 66	<0.0001
*C*-reactive protein (mg/dL)	0.5 ± 2.3	0.4 ± 1.7	0.246
eGFR (ml/min/1.73 m^2^)	91.4 ± 2.6	92.7 ± 2.5	0.311
AST (U/L)	24 ± 12	18 ± 9	<0.0001
ALT (U/L)	25 ± 15	17 ± 10	<0.0001
GGT (U/L)	47 ± 54	31 ± 28	<0.0001
Use of antihypertensive drugs (%)	95.3	91.2	0.652
Use of Lipid lowering drugs (%)	63.6	61.1	0.441

Data are means ± SD. BMI: body mass index; HbA1c: glycated hemoglobin; HOMA-IR, homeostatic model assessment; eGFR: estimated glomerular filtration rate; AST: aspartate aminotransferase; ALT: alanine aminotransferase; GGT: gamma-glutamyl-transpeptidase.

**Table 2:** Energy intake and nutrient composition of the habitual diet in in the clusters NASH or NO-NASH.

	Cluster NASH (n. 642)	Cluster NO-NASH (n. 1384)	*p*-Value
Energy (Kcal/day)	1755 ± 618	1843 ± 692	0.006
Total Proteins (% TEI)	18.4 ± 2.6	18.2 ± 2.6	0.272
Proteins from animal food sources (% TEI)	12.7 ± 3.2	12.5 ± 3.2	0.276
Proteins from vegetable food sources (% TEI)	5.7 ± 1.1	5.7 ± 1.1	0.574
Total Lipids (% TE)	37.1 ± 6.2	36.6 ± 6.1	0.089
Saturated fatty acids (% TEI)	12.2 ± 2.5	12.0 ± 2.5	0.179
Monounsaturated fatty acids (% TEI)	18.3 ± 3.8	18.0 ± 3.8	0.105
Polyunsaturated fatty acids (% TEI)	4.5 ± 1.1	4.5 ± 1.1	0.253
*n*-3 (% TEI)	0.55 ± 0.11	0.55 ± 0.12	0.930
*n*-6 (% TEI)	3.60 ± 1.03	3.56 ± 1.03	0.394
Total cholesterol (mg/1000 kcal/day)	185 ± 53	181 ± 54	0.130
Total Carbohydrates (% TEI)	44.4 ± 7.7	45.1 ± 7.4	0.081
Added sugars (% TEI)	2.37 ± 3.01	2.24 ± 3.24	0.376
Fibre (g/1000 kcal/day)	10.5 ± 2.6	11.0 ± 2.7	<0.0001
Glycemic Index	51.6 ± 3.5	51.9 ± 3.4	0.172
Glycemic Load (%)	105.1 ± 46.6	111.4 ± 50.1	0.019
Alcohol (g/day)	9.9 ± 15.2	11.0 ± 15.3	0.146
Vitamin-C (mg/day)	105 ± 49	115 ± 58	<0.0001
β-carotene (mg/day)	2286 ± 1307	2649 ± 1830	<0.0001
Vitamin E (mg/day)	6.42 ± 2.27	7.00 ± 2.90	<0.0001
Vitamin D (mg/day)	2.47 ± 1.29	2.53 ± 1.53	0.398
TEAC	5.47 ± 2.25	6.00 ± 2.42	<0.0001
TRAP	8.15 ± 3.64	8.91 ± 3.76	<0.0001
FRAP	17.04 ± 7.11	18.46 ± 7.45	<0.0001
Total polyphenols (mg/1000 kcal/day)	377.4 ± 163.1	386.1 ± 165.4	0.026

Data are means ± SD. TEI: total energy intake; TEAC: trolox equivalent antioxidant capacity; TRAP: total radical-trapping antioxidant parameter; FRAP: ferric reducing-antioxidant power.

**Table 3:** Consumption of specific food items (g/1000 Kcal/day), in the clusters NASH or NO-NASH.

	Cluster NASH (n. 642)	Cluster NO-NASH (n. 1384)	*p*-Value
Cereals	97.9 ± 35.1	94.8 ± 36.6	0.075
Pasta	28.2 ± 17.7	26.8 ± 16.3	0.016
Rice	3.55 ± 4.36	3.09 ± 4.04	0.021
Bread	35.6 ± 31.1	36.1 ± 32.6	0.738
Wholegrain Bread	6.7 ± 15.8	8.7 ± 17.6	0.019
Legumes and Nuts	13.6 ± 10.6	14.7 ± 11.8	0.049
Vegetables	95.7 ± 49.6	97.4 ± 49.7	0.047
Potatoes	11.7 ± 12.5	10.1 ± 14.0	0.013
Fruits	151.1 ± 86.1	164.3 ± 92.9	0.002
Meat	73.7 ± 30.7	68.4 ± 29.1	<0.0001
Red meat	56.3 ± 26.8	52.7 ± 25.8	0.004
White meat	1.5 ± 0.9	1.9 ± 0.5	0.001
Processed meat	15.6 ± 11.2	14.0 ± 10.1	0.003
Fish	23.7 ± 18.8	23.0 ± 17.1	0.390
Fatty fish	14.6 ± 12.1	16.3 ± 11.8	0.005
Lean fish	8.5 ± 6.0	8.2 ± 6.5	0.147
Dairy products	100.4 ± 54.9	125.3 ± 60.1	<0.0001
Cheese	20.1 ± 13.0	20.0 ± 13.4	0.965
Milk and Yogurt	80.6 ± 76.0	102.9 ± 88.0	<0.0001
Eggs	10.6 ± 7.2	10.4 ± 7.5	0.543
Vegetable oils	14.0 ± 6.0	13.9 ± 6.0	0.897
Oils from animal origin	1.56 ± 1.54	1.43 ± 1.42	0.067
rMED score	8.1 ± 2.6	9.7 ± 2.5	0.001

Data are means ± SD. rMED: relative Mediterranean Diet.

## Data Availability

Not applicable.
